# Comparison of treatment patterns and economic outcomes in metastatic breast cancer patients initiated on trastuzumab versus lapatinib: a retrospective analysis

**DOI:** 10.1186/2193-1801-3-236

**Published:** 2014-05-08

**Authors:** Annie Guérin, Deepa Lalla, Geneviève Gauthier, Amy Styles, Eric Q Wu, Anthony Masaquel, Melissa G Brammer

**Affiliations:** Analysis Group, Inc., 1000 rue de la Gauchetière Ouest, Bureau 1200, Montreal, QC H3B 4W5 Canada; Genentech, Inc., South San Francisco, CA USA

**Keywords:** Metastatic breast cancer, Trastuzumab, Lapatinib

## Abstract

Few studies have compared treatment patterns, healthcare resource utilization (HRU), and costs in patients with metastatic breast cancer (mBC) receiving HER2 directed therapy. This study evaluated these outcomes in patients receiving trastuzumab or lapatinib. Adult women with mBC, who were initiated on trastuzumab or lapatinib, on or after March 13, 2007, were selected from the US-based PharMetrics® Integrated Database (2000–2011). Patients were required to be continuously enrolled in their healthcare plan for ≥6 months prior to and ≥30 days following trastuzumab or lapatinib initiation. Trastuzumab or lapatinib discontinuation rates (defined as a gap ≥45 consecutive days) were compared using multivariate Cox proportional-hazards models. HRU and monthly healthcare cost differences were estimated using multivariate negative binomial regression models and generalized linear models, respectively. Among the 643 patients who met the inclusion criteria, 381 and 262 patients were included in the trastuzumab and lapatinib groups, respectively. The majority of the 262 patients receiving lapatinib previously received trastuzumab (N = 171 [65.3%]). After adjustment for potential confounders, when compared to trastuzumab patients, lapatinib patients had a higher rate of treatment discontinuation (hazard ratio [HR] = 1.57; *P* < 0.001), a higher rate of outpatient visits (not treatment administration related) (IRR = 1.19; *P* < 0.004), and a lower rate of medical visits associated with treatment administration (IRR = 0.34; *P* < 0.001). There were no significant differences between the two groups in total monthly healthcare costs ($11,920 vs. $11,898 for trastuzumab and lapatinib patients, respectively; *P* = 0.451). Findings from our study show that, irrespective of the treatment initiated at index date, disease management in patients with mBC is associated with similar and substantial healthcare costs. Any differences in specific components of healthcare costs were associated with differences in the mode of treatment administration. Approximately 50% of all costs were non-drug related, and future studies should focus on how these costs may be controlled, regardless of mode of treatment administration.

## Introduction

Metastatic breast cancer (mBC) is the second most fatal cancer among women (Susan G Komen Foundation 2013). In 2014, 232,670 women are expected to be diagnosed with invasive breast cancer in the United States (Siegel et al. 2014), and approximately 30% of all cases of primary breast cancer are known to eventually metastasize (Mayer 2011). Economically, mBC is known to impose a substantial burden. Studies reporting the cost of mBC treatment have noted costs ranging from ~ $7,500 per month (calculated using data collected between 2000 and 2006) to ~ $10,000 per month (calculated in 2010 US dollars) (Vera-Llonch et al. 2011; Montero et al. 2012).

The human epidermal growth factor receptor 2 (HER2) protein is a common marker in mBC detection; the HER2 protein is known to signal cell growth and division and thus to promote the growth of cancer cells. HER2-positive tumors have been implicated in mBC (Guy et al. 1992), with approximately 20% to 25% of all patients having tumors that are HER2-positive (Slamon et al. 1987, 1989). In addition, HER2-positive tumor status has been associated with more aggressive disease, increased disease recurrence, and a poor response to regular chemotherapy (Borg et al. 1990; Kumar et al. 2007; Gullo et al. 2013).

Treatment goals for patients with mBC are to prolong patient survival, and to minimize cancer-associated disease symptoms, allowing stability or improvement in the patient’s quality-of-life. Prior to 2012, biological therapy, including trastuzumab (Herceptin™, Genentech, Inc., South San Francisco, CA, USA) and lapatinib (Tykerb™, GlaxoSmithKline, LLC, London, UK), was the primary treatment option for patients with HER2-positive mBC. Trastuzumab is a HER2 antagonist that binds directly to the protein receptor, and is recommended in combination with paclitaxel for first line treatment, or as a single agent in patients who have received one or more chemotherapy regimens for metastatic disease (Slamon et al. 2001). Trastuzumab has been shown to increase the clinical benefit of first-line chemotherapy (Slamon et al. 2001). The other commonly used biologic, lapatinib, is a tyrosine kinase inhibitor that interrupts HER2 pathways, and is recommended in combination with capecitabine in patients who have received prior chemotherapy or in combination with letrozole in postmenopausal women with hormone-receptor positive metastatic breast cancer for whom hormonal therapy is indicated (Medina and Goodin 2008; GlaxoSmithKline, LLC 2007). The National Cancer Commission Network (NCCN) guidelines recommend trastuzumab or lapatinib as the preferred treatment agents for recurrent or metastatic HER2-positive BC (National Comprehensive Cancer Network 2012). Administration of the two agents differs, as lapatinib is orally-administered, while trastuzumab must be administered by a healthcare professional as an intravenous infusion (GlaxoSmithKline, LLC 2007; Genentech, Inc. 2006).

No studies have compared the clinical and economic outcomes associated with trastuzumab- versus lapatinib-based treatments in mBC in a real-world setting. Given that trastuzumab and lapatinib have different mechanisms of action and administration, it is unclear whether these two drugs will be associated with similar treatment patterns, healthcare resource utilization (HRU), and healthcare costs. The objective of this study is, therefore, to compare treatment discontinuation, HRU, and healthcare costs among patients with mBC initiated on trastuzumab versus lapatinib in a real world setting.

## Patients and methods

### Data source

The study was conducted using data from the PharMetrics® Integrated Database (PharMetrics) from 2000–2011. The PharMetrics database comprises medical and drug claims for more than 70 million members from over 100 healthcare plans, covering all census regions of the United States, with a concentration in the southern and midwestern regions. Data contained within the database include patient demographics, healthcare plan enrollment, diagnoses and procedures, and drug prescription dispensing claims (PharMetrics 2011). Data are de-identified and comply with the patient requirements of the Health Insurance Portability and Accountability Act (HIPAA). Therefore, Institutional Review Board approval was not required.

### Patient selection and study design

**Figure 1:**
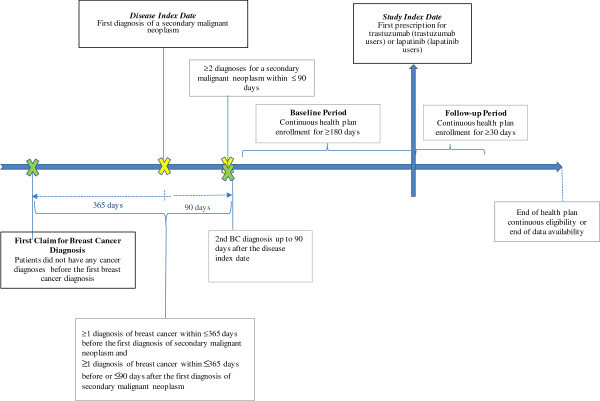
**Sample selection**.

Eligible patients were included in the study if they: (1) initiated trastuzumab or lapatinib on or after March 13, 2007 (the FDA approval date of lapatinib); (2) had at least two independent secondary malignant neoplasm diagnoses (ICD-9-CM: 197.xx, 198.xx) within 90 days, occurring prior to or on the date of trastuzumab or lapatinib initiation; (3) had at least two BC diagnoses (ICD-9-CM: 174.x) during the 365 days prior to, and up to 90 days following, the first mBC diagnosis — with at least one during the 365 days prior to the first mBC diagnosis; and (4) were continuously enrolled in their healthcare plan for at least 6 months prior to, and at least 30 days following, the trastuzumab or lapatinib initiation date. Patients with mBC were excluded from the study if they had a diagnosis for any other cancer prior to the first BC diagnosis, or of if they used trastuzumab and lapatinib concomitantly within the first 28 days following treatment initiation (Figure 1).

Patients who met the above sample selection criterion were classified into two mutually exclusive cohorts based on the first drug initiated on or after March 13, 2007 (the FDA approval date for lapatinib): (1) trastuzumab patients; and (2) lapatinib patients. The first prescription fill date/therapy administration date was defined as the index date.

A retrospective intention-to-treat cohort design was used to compare outcomes between patients who initiated trastuzumab and lapatinib. The baseline period was defined as the 6-month period prior to the index date. The study period spanned from the index date up to the end of healthcare plan enrollment or the end of data availability, whichever occurred first.

### Outcomes and statistical analyses

#### Measures and study outcomes

*Patient characteristics*. Baseline characteristics included the following: demographic data (age and geographic region); index year; time from the first mBC diagnosis to the index date; the Charlson Comorbidity Index (CCI) findings (Quan et al. 2005); physical and mental comorbidities (Elixhauser et al. 1998); prior cancer treatments; HRU; and costs at baseline. Study period characteristics included the number of days between the index date and the end of the study period; the number of days between the index date and treatment discontinuation or the end of the study, whichever occurred first; and cancer treatments used during the study period.

*Treatment discontinuation* was defined as the first interruption of the index drug for at least 45 consecutive days (the interruption may have occurred between the end of one prescription fill/infusion and the start of the next prescription fill/infusion, or between the end of one prescription fill/infusion and the end of the study period). The discontinuation date was defined as the last day of supply before the treatment discontinuation.

*HRU* during the study period was identified using medical claims, and was reported for the following categories: inpatient admission, inpatient days, emergency room visits, outpatient visits, medical services associated with treatment administration, and other medical services (e.g. laboratory, radiology, ancillary services). The category “medical services associated with treatment administration” was defined as medical visits associated with a procedure for treatment administration by a health care professional (e.g., injection or infusion of medications).

*Healthcare costs* incurred during the study period were reported from a payer’s perspective. As the duration of the observation periods varied across patients, costs were reported as monthly costs. Healthcare costs were adjusted for inflation using the consumer price index for medical categories and expressed in 2012 U.S. dollars. Healthcare cost categories included inpatient costs, outpatient costs, emergency room costs, other medical service costs, and total medical costs. We also examined medical costs associated with treatment administration, drug-only cost, and total drug costs. Total healthcare costs were defined as the sum of total medical costs and total drug costs.

#### Sensitivity analysis (prior trastuzumab patients)

A large proportion of lapatinib patients (65.3%) were treated with trastuzumab prior to the index date, which was either in the adjuvant or metastatic setting. This may indicate that lapatinib patients have a different severity and treatment profile. Accordingly, a sensitivity analysis was performed in which lapatinib patients were stratified based on prior trastuzumab use into two mutually exclusive cohorts: (1) lapatinib patients *with* prior trastuzumab use; and (2) lapatinib patients *without* prior trastuzumab use. Each cohort was compared to trastuzumab patients regarding each of the above study outcomes.

#### Statistical analyses

Patient characteristics were compared using Wilcoxon rank-sum tests for continuous variables and chi-square tests for categorical variables.

Discontinuation rates for lapatinib and trastuzumab patients were estimated using Kaplan-Meier survival analyses and were then compared between the two cohorts using log-rank tests. In addition, multivariate Cox proportional hazard models were used to compare treatment discontinuation between the two cohorts, while adjusting for differences in confounding factors. Results were reported as adjusted hazard ratios (HRs) with their 95% confidence intervals (CIs) and *P*-values.

Incidence rates from each HRU category were reported per patient-year for the two cohorts. In addition, generalized linear models (GLM) with a log link and a negative binomial distribution were used to compare HRU between lapatinib and trastuzumab patients while adjusting for differences in confounding factors. Results were reported as adjusted incidence rate ratios (IRRs) with *P*-values.

Average monthly healthcare costs were compared between lapatinib and trastuzumab patients using Wilcoxon rank-sum tests. In addition, adjusted (for confounding factors) cost differences between lapatinib and trastuzumab patients were estimated using GLM with a log link and gamma distribution or were calculated using two-part models for cost categories when > 5% of the observations had a cost of $0 (where the first part is a logistic model and the second part is a GLM model with a log link and a gamma distribution). *P*-values were estimated using non-parametric bootstrap resampling techniques of 499 iterations.

For all outcomes, multivariate analyses were used to adjust for potential confounding factors. Covariates were selected based on baseline variables with statistically significant differences between the two study cohorts, and with at least 5% prevalence in each cohort, and included baseline demographics (age, region of residence), index year, chemotherapy or anti-angiogenic use during the baseline period, radiation therapy use during the baseline period, baseline HRU, and time from first mBC diagnosis to index date.

All statistical analyses were carried out with SAS 9.2 statistical software (SAS Institute, Inc., Cary, NC, USA).

## Results

### Patient characteristics

**Figure 2:**
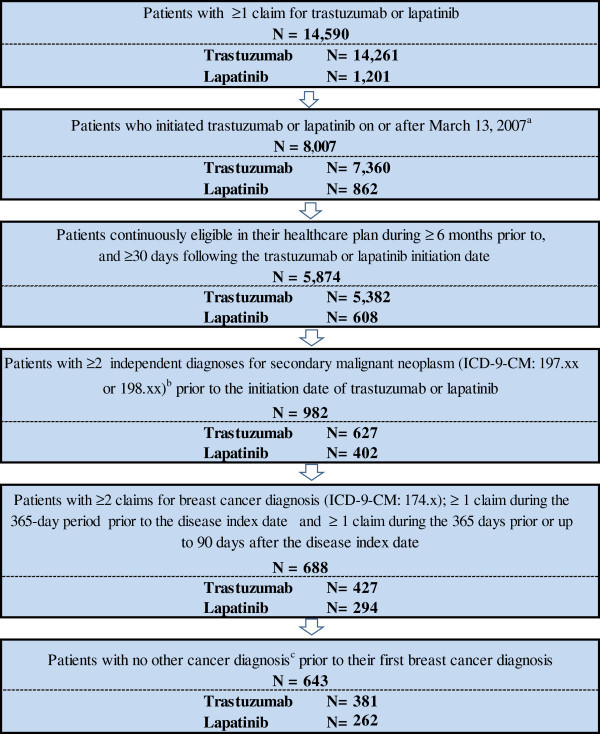
**Sample selection flow chart**.

Overall, 643 patients met the inclusion criteria, of which 381 initiated trastuzumab, and 262 initiated lapatinib (Figure 2). Trastuzumab patients were older than lapatinib patients (56.7 vs. 54.4; *P* = 0.020), and a greater proportion of trastuzumab patients were from the southern region of the United States (39.6% vs. 28.2%; *P* = 0.003), while a greater proportion of lapatinib patients were from the midwestern area (38.5% vs. 30.7%; *P* = 0.039; Table 1). Trastuzumab patients had a shorter time from first mBC diagnosis to index date (185.8 days vs. 518.5 days; *P* < 0.001). A greater proportion of lapatinib patients received chemotherapy or anti-angiogenic therapy during the baseline period (29.9% vs. 58.8%, *P* < 0.001) and had used chemotherapy or biologic therapy between the first mBC diagnosis and the index date (71.8% vs. 31.0%; *P* < 0.001). The majority (65.3%) of lapatinib patients had used trastuzumab prior to the index date.

**Table 1:** **Baseline characteristics: demographics and prior therapies**

Characteristics	Trastuzumab	Lapatinib	***P***-value
Total number of patients	N = 381	N = 262	
Age at index date (mean ± SD)	56.7 ± 12.0	54.4 ± 10.2	0.020**
Region, N (%)			
East	74 (19.4)	63 (24.0)	0.159
Midwest	117 (30.7)	101 (38.5)	0.039**
South	151 (39.6)	74 (28.2)	0.003**
West	39 (10.2)	24 (9.2)	0.652
Index year, N (%)			
2007	101 (26.5)	98 (37.4)	0.003**
2008	131 (34.4)	78 (29.8)	0.220
2009	89 (23.4)	46 (17.6)	0.076
2010	60 (15.7)	40 (15.3)	0.869
Time from first mBC diagnosis to index date (days; mean ± SD)	185.8 ± 329.4	518.5 ± 490.6	< 0.001**
Prior cancer treatments, N (%)			
Trastuzumab use, any time prior to the index date	-	171 (65.3)	
Hormonal therapy, during the baseline period	104 (27.3)	61 (23.3)	0.252
Radiation therapy, during the baseline period	104 (27.3)	103 (39.3)	0.001**
Surgery (mastectomy), during the baseline period	61 (16.0)	7 (2.7)	< 0.001**
Chemotherapy or anti-angiogenic therapy, during the baseline period	114 (29.9)	154 (58.8)	< 0.001**
Chemotherapy or biological therapy, between the first mBC diagnosis and index date	118 (31.0)	188 (71.8)	< 0.001**
CCI^a^ (mean ± SD)	6.7 ± 1.0	6.6 ± 1.2	0.224
Physical comorbidities^b^, N (%)			
Other neurological diseases	22 (5.8)	28 (10.7)	0.022**
Chronic pulmonary diseases	49 (12.9)	19 (7.3)	0.023**
Iron deficiency anemia	64 (16.8)	70 (26.7)	0.002**

**Denotes statistical significance at the 5% level.

^a^CCI, Charlson Comorbidity Index.

^b^Only comorbidities with at least 5% prevalence in each cohort that were statistically significantly different between lapatinib and trastuzumab patients were reported.

N, number of patients; SD, standard deviation.

With respect to comorbidities during the baseline period, trastuzumab patients had a higher prevalence of chronic pulmonary diseases (12.9% vs. 7.3%; *P* = 0.023), and lapatinib patients had a higher prevalence of neurological diseases (10.7% vs. 5.8%; *P* = 0.022) and anemia (26.7% vs. 16.8%; *P* = 0.002).

During the baseline period, trastuzumab patients had fewer outpatient visits and lower associated costs (22.0 vs. 26.3, *P* < 0.001; $16,662 vs. $26,572, *P* = 0.006), fewer medical visits and costs associated with treatment administration (5.1 vs. 7.6, *P* < 0.001; $6,153 vs. $16,179, *P* < 0.001), lower total drug costs ($1,840 vs. $4,598, *P* < 0.001), and lower total healthcare costs ($38,655 vs. $64,261, *P* < 0.001).

Compared to lapatinib patients, trastuzumab patients had a longer treatment duration (i.e., the number of days between the index date and treatment discontinuation or the end of the study period) (269.1 days vs. 179.8 days; *P* < 0.001) (Table 2). A greater proportion of trastuzumab patients were prescribed hormonal therapy (28.9% vs. 9.5%; *P* < 0.001), and underwent mastectomy (8.7% vs. 0.8%; *P* < 0.001; Table 2) over the study period. The majority of trastuzumab (84.0%) and lapatinib patients (87.0%) were co-prescribed chemotherapy or anti-angiogenic agents during the study period.

**Table 2:** **Description of therapies used during the study period: trastuzumab patients versus lapatinib patients**

	Trastuzumab	Lapatinib	***P***-value
	N = 381	N = 262	
Number of days between the index date and the end of the study period, mean ± SD	453.3 ± 334.0	412.9 ± 327.1	0.123
Treatment duration (i.e. the number of days between the index date and treatment discontinuation or the end of the study period), mean ± SD	269.1 ± 235.1	179.8 ± 189.0	< 0.001**
Cancer treatments used during the study period, N (%)			
Hormonal therapy	110 (28.9)	25 (9.5)	< 0.001**
Radiation therapy	168 (44.1)	114 (43.5)	0.884
Surgery (mastectomy)	33 (8.7)	2 (0.8)	< 0.001**
Chemotherapy or anti-angiogenic therapy, N (%)			
Any chemotherapy or anti-angiogenic therapy^a^	320 (84.0)	228 (87.0)	0.287
Capecitabine	71 (18.6)	194 (74.0)	< 0.001**
Paclitaxel	156 (40.9)	48 (18.3)	< 0.001**
Docetaxel	112 (29.4)	21 (8.0)	< 0.001**
Carboplatin	103 (27.0)	25 (9.5)	< 0.001**
Vinorelbine	86 (22.6)	40 (15.3)	0.022**
Gemcitabine	51 (13.4)	26 (9.9)	0.184
Bevacizumab	29 (7.6)	28 (10.7)	0.178
Doxorubicin	38 (10.0)	21 (8.0)	0.398
Cyclophosphamide	39 (10.2)	12 (4.6)	0.009**
Fluorouracil	17 (4.5)	5 (1.9)	0.080
Ixabepilone	15 (3.9)	15 (5.7)	0.291
Epirubicin	8 (2.1)	0 (0.0)	-
Cisplatin	2 (0.5)	3 (1.1)	0.379
Mitoxantrone	1 (0.3)	1 (0.4)	0.790
Etoposide^b^	0 (0.0)	0 (0.0)	-
Vinblastine^b^	0 (0.0)	0 (0.0)	-
Any chemotherapy or anti-angiogenic therapy within 28 days following the index date	285 (74.8)	193 (73.7)	0.745

**Denotes statistical significance at the 5% level.

^a^Any chemotherapy or anti-angiogenic therapy during the study period or at the index date.

^b^Neither etoposide nor vinblastine were prescribed during the study period.

N, number of patients; SD, standard deviation.

### Study outcomes

**Figure 3:**
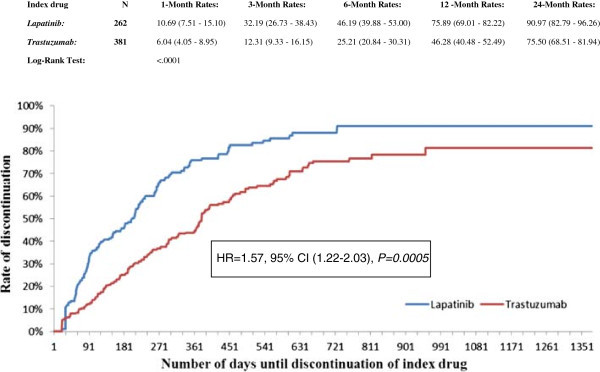
**Comparison of discontinuation rates between patients initiated on trastuzumab and patients initiated on lapatinib**.

During the study period, 63.4% of lapatinib patients and 48.3% of trastuzumab patients discontinued treatment. After adjusting for confounders, lapatinib patients had a risk of treatment discontinuation that was 57% higher than that of trastuzumab patients (HR 1.57, 95% CI 1.22-2.03; *P* < 0.001; Figure 3).

**Figure 4:**
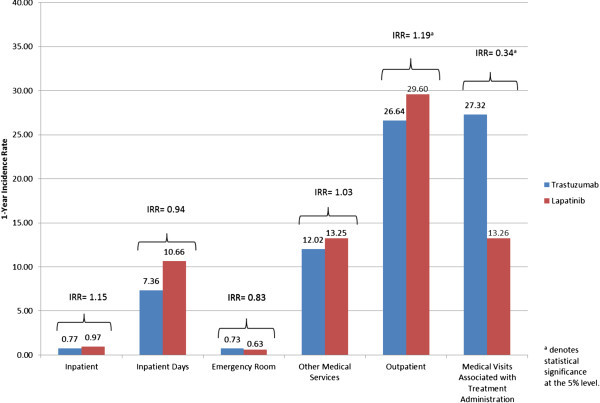
**One-year incidence rates for each category of HRU**.

After adjusting for confounders, lapatinib patients had a higher incidence of outpatient visits (IRR 1.19, 95% CI 1.06-1.34; *P* = 0.004; Figure 4), and a lower incidence of medical visits that were associated with treatment administration (IRR 0.34, 95% CI 0.29-0.41; *P* < 0.001; Figure 4). There were no statistically significant differences found for inpatient, emergency room, or other medical service visits between trastuzumab patients and lapatinib patients.

**Figure 5:**
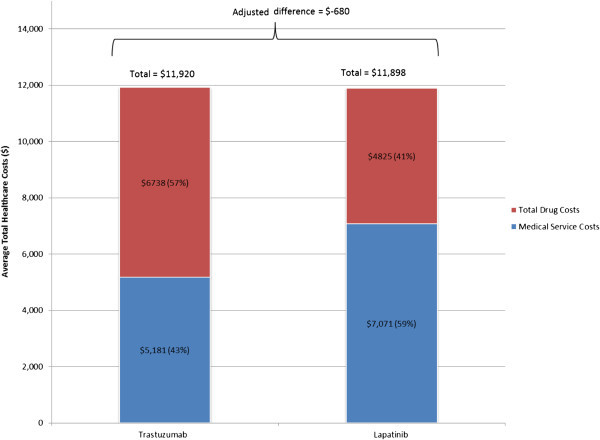
**Average monthly total healthcare costs**.

**Figure 6:**
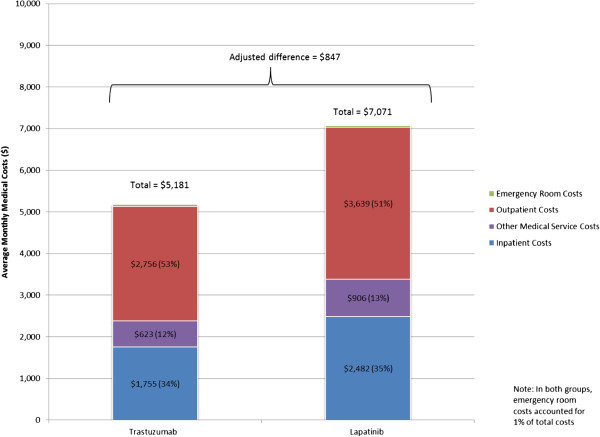
**Average monthly total medical costs**.

**Figure 7:**
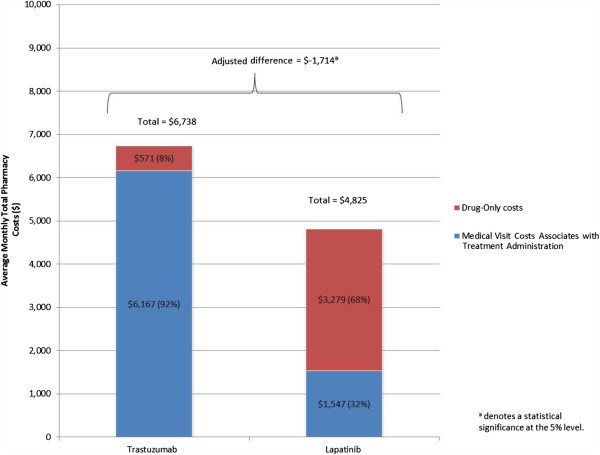
**Average monthly total drug costs**.

After adjusting for confounders, results showed that overall there was no statistically significant difference in the total healthcare cost incurred by lapatinib versus trastuzumab patients (Figure 5). On average, patients in both cohorts had a total monthly healthcare cost of approximately $12,000. Although lapatinib patients incurred higher outpatient costs ($3,639 vs. $2,756, adjusted difference = $847; *P* = 0.104; Figure 6) and drug-only costs ($3,279 vs. $571, adjusted difference = $2,676; *P* < 0.001; Figure 7), this was balanced by the higher medical cost associated with treatment administration incurred by trastuzumab patients ($6,167 vs. $1,547, adjusted difference = $4,904; *P* < 0.001; Figure 7). This higher medical cost for treatment administration led to higher total drug costs among trastuzumab patients ($6,738 vs. $4,825, adjusted difference = $1,714; *P* < 0.001; Figure 7). There was no significant difference in total medical service costs, inpatient costs, emergency room costs, and other medical service costs between study cohorts (Figure 6).

### Sensitivity analysis

Overall, findings from the sensitivity analysis were consistent with the main analysis (results not reported). Few differences were found for HRU and healthcare costs, wherein (1) lapatinib patients *without* prior trastuzumab use had a higher incidence of inpatient admissions (IRR 1.74, *P* = 0.002) compared to trastuzumab patients; (2) the comparison between lapatinib patients *with* prior trastuzumab use and trastuzumab patients was no longer statistically significant for outpatient visits; and (3) lapatinib patients *without* prior trastuzumab use experienced higher medical service costs when compared to trastuzumab patients by $3,825 (*P* < 0.001).

## Discussion

Findings from this study indicate that mBC patients incurred substantial healthcare costs irrespective of the index treatment (i.e., lapatinib or trastuzumab). Overall, patients incurred an average monthly total healthcare cost of approximately $12,000 per patient per month. This finding is consistent with previous studies that reported average healthcare costs ranging from ~ $7,500 per month to ~ $10,000 per month (Kumar et al. 2007; Gullo et al. 2013). In our study, although the two groups had similar total monthly costs, the cost drivers for lapatinib and trastuzumab patients were different: while 57% of trastuzumab user costs resulted from total drug costs, 59% of lapatinib user costs resulted from total medical service costs. Non-drug costs accounted for approximately half of the total costs (43% and 59% for trastuzumab and lapatinib patients, respectively), and, although total drug costs were greater among trastuzumab patients due to treatment administration costs, total costs were not statistically significantly different among the two patient groups due to the higher medical service costs incurred by lapatinib patients. Lapatinib and trastuzumab patients were also generally comparable in terms of HRU, except for the incidence of outpatient visits, which was higher in lapatinib patients, and the incidence of medical visits associated with treatment administration, which was higher in trastuzumab patients. The higher costs for medical visits associated with treatment administration in trastuzumab patients are mainly explained by the method of administration of trastuzumab, which must be administered by a healthcare professional as an intravenous infusion.

The findings of this study also show that trastuzumab patients were more persistent in continuing treatment than the patients receiving lapatinib. Improved treatment persistence, which is essential to treatment adherence, may be somewhat surprising in this case, as lapatinib is an oral medication and therefore presumably convenient to take. Trastuzumab patients may, however, be more persistent because of closer clinical follow-up, as clinical visits are required for drug administration. Further analyses are warranted to better understand the reasons for discontinuation and the factors impacting persistence among patients treated with trastuzumab versus lapatinib.

The FDA recently approved two new therapies for HER2-positive mBC: (1) pertuzumab (Perjeta™, Genentech, Inc.), a HER2 receptor antagonist, indicated in combination with trastuzumab and docetaxel in HER2-positive patients who have not received prior anti-HER2 therapy or chemotherapy for metastatic disease (Genentech, Inc. 2012); and (2) ado-trastuzumab emtansine (Kadcyla™, Genentech, Inc.), a HER-targeted antibody and microtubule inhibitor conjugate, indicated as a single agent for the treatment of patients with HER2-positive mBC who have received prior therapy for mBC or who have developed disease recurrence within 6 months of completing adjuvant therapy (Genentech, Inc. 2013). Due to their recent approval at the time the study was conducted and the limited data availability, these two mBC therapies were not included in the scope of this study.

This study was subject to common limitations of retrospective administrative database studies. First, lapatinib patients may have been more severely ill, given their prior trastuzumab use and longer time to initial treatment of targeted therapy; however, the claims database did not contain information on disease severity. We attempted to control for severity by adjusting for observable variables, such as the time between the first mBC diagnosis and the index date, comorbidities, baseline healthcare utilization and costs, and the use of other cancer treatments prior to the index date. Prior trastuzumab use may also be an indicator of disease severity, and, consequently, we investigated this possibility by stratifying patients based on prior trastuzumab use.

Retrospective databases are also subject to coding errors or data omissions; however, these are expected to affect all treatment cohorts to a similar extent and are unlikely to alter conclusions.

An additional limitation of this study is that it did not distinguish between the patient’s lines of therapy (first, second, third, etc.). The ideal cost and HRU comparison would be made between trastuzumab patients and lapatinib patients without prior therapy use; however the sample size was too restrictive. Therefore, we conducted a sensitivity analysis stratified by prior trastuzumab use, and found robust results. Sensitivity analyses comparing outcomes between lapatinib patients with and without prior trastuzumab use versus trastuzumab patients demonstrated that results were generally consistent with those from the main analysis (i.e., regardless of prior trastuzumab use, lapatinib use was associated with similar HRU, total healthcare costs, and greater treatment discontinuation). Prior trastuzumab use seems to be associated with an unexpected benefit, since lapatinib patients *without* prior trastuzumab treatment experienced a greater number of inpatient visits, while the same effect was not observed for lapatinib patients *with* prior trastuzumab use. Lapatinib patients *without* prior trastuzumab use were also found to have higher medical service costs, as compared to trastuzumab patients. Finally, the study sample was limited to privately insured employees or their dependents diagnosed with mBC, and who whom the main subscriber remained employed for the duration of the study period; thus, these findings may not be generalizable to the overall population of patients with mBC. Nonetheless, claims data remain a valuable source of information, as they comprise a valid, large sample reflecting patients’ behavior in a real-world setting.

In conclusion, findings from our study show that, irrespective of the treatment initiated at the index date, disease management in patients with mBC is associated with similar and substantial healthcare costs in both cohorts, with the only observed healthcare cost differences stemming from differences in mode of treatment administration, and with non-drug costs accounting for an average of 48.5% of total costs. Patients initiated on lapatinib had higher rates of treatment discontinuation, more outpatient visits not related to treatment administration, and fewer medical visits associated with treatment administration. Prior trastuzumab use in lapatinib patients suggests that lapatinib patients may have experienced a more severe disease course. However, results from the sensitivity analyses that take into account this prior use were generally consistent with findings from the main analysis and do not change the overall study conclusion.
